# Hypovitaminosis D in chronic kidney disease

**DOI:** 10.1590/2175-8239-JBN-2021-S106

**Published:** 2021-12-03

**Authors:** Sérgio Gardano Elias Bucharles, Fellype Carvalho Barreto, Rodrigo Bueno de Oliveira

**Affiliations:** 1 Universidade Federal do Paraná, Medical Clinic Department, Service of Nephrology, Curitiba, PR, Brazil.; 2 Universidade Federal do Paraná, Hospital de Clínicas Complex, Service of Nephrology, Curitiba, PR, Brazil.; 3 Universidade de Campinas, Faculdade de Ciências Médicas, Medical Clinic Department, Service of Nephrology, Campinas, SP, Brazil.

## Recommendations

1. Vitamin D levels should be assessed in patients with CKD G3A-5D at the beginning of clinical follow-up due to the high prevalence of hypovitaminosis D in this population and its association with secondary hyperparathyroidism (SHPT) and reduced bone mass (Evidence).

2. The assessment frequency of vitamin D serum levels should be individualized, depending on baseline levels and therapeutic intervention.

3. Vitamin D supplementation (ergocalciferol or cholecalciferol) for patients with CKD G3A-5D and post renal transplantation should be implemented in the presence of serum vitamin D levels lower than 30 ng/mL (Evidence).

3.1 Vitamin D supplementation should not be started in the presence of hypercalcemia and until this condition is corrected (Evidence).

3.2 During vitamin D supplementation, calcium, phosphorus and PTH levels should be assessed as recommended for the CKD stage (Evidence). 

3.3 Supplementation should be discontinued if the patient develops hypercalcemia (Ca > 10.5 mg/dL) and/or 25(OH)D levels > 100 ng/mL (Evidence). 

3.4 Supplementation should be maintained at least until serum vitamin D levels normalize (Opinion).

## Rational

Vitamin D is naturally produced endogenously, as vitamin D3, from cholesterol. In the skin, 7-dehydrocholesterol is converted into pre-vitamin D by ultraviolet action, and subsequently undergoes thermo isomerization to be converted into vitamin D3^1^. On the other hand, vitamin D2 (ergocalciferol) is derived from plant ergosterol, obtained mainly from diet. Under normal conditions, about 20-30% of the natural form of vitamin D is obtained through nutrition, either vitamin D2 or vitamin D3. There are few foods with good amounts of vitamin D (fatty fish, fish oil, eggs). Thus, skin exposure to the sunlight is essential for maintaining adequate levels of vitamin D^2^ ^,^ ^3^. Natural forms of vitamin D (D2 and D3) are transported to the liver by the vitamin D-binding protein and both are hydroxylated at their carbon 25 and converted to 25(OH)D (calcidiol or calcifediol) by several different 25-hydroxylases^4^.

The circulating level of 25(OH)D is considered the most useful marker of vitamin D stores in the body^1^ ^-^ ^4^. Kidneys are essential for maintaining adequate serum levels of vitamin D, due to uptake from the glomerular ultrafiltrate and subsequent recirculation^5^. Additionally, 25(OH)D taken up by the kidneys is converted into its active form (calcitriol) by the action of renal 1-alpha hydroxylase (CYP27B1), which activity is stimulated by parathyroid hormone (PTH) and suppressed by fibroblast growth factor 23 (FGF23) and by calcitriol itself^4^. In addition, several other extra-renal tissues and cell types have the necessary enzymatic armamentarium (megalin and 1-alpha hydroxylase) for local conversion of calcitriol, especially the main cells of the parathyroid glands, osteoblasts, digestive tract, endothelium, cardiomyocytes, and immune system^6^, sites where vitamin D can exert its traditional (regulation of parathyroid activity, control of calcium and phosphorus balance, bone mineralization) or non-traditional (pleiotropic effects) functions^6^. 

Both previous national^7^ and international^8^ ^,^ ^9^ ^,^ ^10^ guidelines suggest that serum 25(OH)D levels should be assessed in patients with chronic kidney disease (CKD) G3A-5D and that correction of hypovitaminosis D (25(OH)D levels < 30.0 ng/mL) should be done. Individuals with serum levels ≤ 20.0 ng/mL are classified as vitamin D deficient, while values between 20.1-29.9 ng/mL, as insufficient^11^.

Patients with CKD in its various stages^12^ ^,^ ^13^, especially among dialysis patients^14^ ^,^ ^15^ and the kidney transplant population^16^, present high prevalence of hypovitaminosis D. Table 1 shows the main causes and risk factors for hypovitaminosis D in the CKD population. 

**Table 1.** Main causes and risk factors for hypovitaminosis D in CKD

Advanced Age
Female gender
Obesity
Proteinuria
Diabetes *mellitus*
Peritoneal dialysis
Reduced expression of vitamin D receptor
Impaired tubular reabsorption of 25(OH)D
Reduction in cutaneous synthesis of 25(OH)D
Use of calcineurin inhibitors
Reduced hepatic synthesis of 25(OH)D

Source: Adapted from Souberbielle and Chazot, 2017^11^.

In CKD, low vitamin D levels are associated with secondary hyperparathyroidism (SHPT), high turnover bone disease, and reduced bone mineral density^17^ ^-^ ^20^. Additionally, the presence of hypovitaminosis D is associated with muscle weakness and falls in hemodialysis patients^21^, besides metabolic syndrome and obesity^22^, left ventricular hypertrophy^23^ and vascular calcifications^24^ ^,^ ^25^. Furthermore, hypovitaminosis D is associated with early mortality in incident patients on hemodialysis therapy^26^, anemia^27^, systemic inflammation^14^ ^,^ ^28^ and albuminuria^28^. A meta-analysis study identified that each 10 ng/mL increase in 25(OH)D levels was associated with a 14% reduction in mortality risk among CKD patients^29^.

Finally, although poorly documented in clinical trials, it is suggested that renoprotective effects of vitamin D may be linked to inhibition of the renin-angiotensin-aldosterone system and the NF-kβ pathway^30^, in addition to increased nitric oxide synthesis by vascular endothelium^31^. 

## Nutritional vitamin D in patients with CKD G3-5

Although there is controversy in medical literature, it is postulated that patients with CKD G3-5 should have serum 25(OH)D levels known and maintained above 30 ng/mL, in order to prevent SHPT and reduce the risk of fragility fractures^7^ ^,^ ^8^. Additionally, the most recent KDIGO 2017 guideline recommends assessing 25(OH)D levels in CKD G3-4 when PTH values are progressively increasing or persistently above the upper limit of normality, suggesting correction of hypovitaminosis D for these cases, without, however, considering a reference value for vitamin D^10^. Table 2 compiles the information proposed by the main guidelines on investigation and therapeutic management for non-dialytic CKD patients. In advanced CKD, 25(OH)D is converted into calcitriol due to the extra-renal production of this hormone^32^, which would justify the use of native vitamin D supplementation as an auxiliary tool in mitigating calcitriol deficiency^33^. 

Traditionally, there are three available forms for 25(OH)D replacement: two prodrugs (cholecalciferol and ergocalciferol), which require conversion by hepatic 25-alpha-hydroxylase to form 25(OH)D3 and 25(OH)D2, respectively; and calcifediol, available as 25(OH)D3^3^. Several clinical studies suggest the superiority of cholecalciferol over ergocalciferol in determining increased 25(OH)D levels^34^, being suggested as the first choice for supplementation. 

Although PTH target values for the population with CKD 3-5 are not well defined to date, the use of the vitamin D nutritional form is suggested as initial measure for prevention and treatment of SHPT^3^ ^,^ ^35^. A meta-analysis including four randomized clinical trials that compared the effects of nutritional vitamin D versus placebo in patients with non-dialytic CKD suggested that supplementation of vitamin D, cholecalciferol or ergocalciferol, is able to increase serum 25(OH)D levels and reduce PTH levels^3^ ^,^ ^36^ ^,^ ^37^. Additionally, higher doses of 25(OH)D3 (cholecalciferol 50,000 IU per week for 12 weeks, followed by 50,000 IU per week every 2 weeks for 40 weeks) were associated with more pronounced and longer-lasting reductions in PTH, besides stability of 25(OH)D levels^38^. Treatment should be discontinued in the presence of 25(OH)D levels > 100 ng/mL and/or serum calcium > 10.5 mg/dL in the absence of concomitant treatment with active forms of vitamin D3.

**Table 2.** Guidelines and nutritional vitamin D in CKD 3-5 (not on dialysis)

Guideline	25(OH)D Assessment	25(OH)D Target	Supplementation	Therapeutic Indication
KDOQI 2003	In the presence of PTH > upper limit of the method	≥ 30 ng/mL	6 months with ergocalciferol	First-line treatment of SHPT
KDIGO 2009	Initially and during treatment in CKD 3-5	Same recommendation as general population	-	First-line treatment of SHPT
NICE^*^ 2014	All patients with CKD 4-5	≥ 20 ng/mL	-	Treating hypovitaminosis D and SHPT
KDIGO 2017	All patients with CKD 3-5 in the presence of PTH > upper limit of the method or progressively elevated	Same recommendation as general population (no proposed level)	-	Treatment of SHPT in conjunction with corrective actions on calcemia, phosphatemia and dietary phosphorus intake

* NICE: National Institute of Clinical Excellence

## Nutritional Vitamin D in patients with CKD G5D

Hypovitaminosis D is common in the chronic dialysis population. Particularly in hemodialysis patients, low 25(OH)D levels have been associated with early mortality in incident patients^26^ ^,^ ^39^, late overall mortality^40^ and all-cause mortality (cardiovascular, infectious, and oncological)^41^. Two previous guidelines (International Osteoporosis Foundation 2010 and KDOQI 2003)^8^ ^,^ ^42^ suggest that for CKD 3-5 adult patients, optimal 25(OH)D values should be > 30 ng/mL. This same therapeutic target has been extrapolated to the population with CKD 5D. In several prospective observational studies, administration of nutritional vitamin D, in varied amounts and frequency, resulted in a significant increase in 25(OH)D levels, with no considerable impact on other mineral metabolism parameters^43^ ^-^ ^46^, cholecalciferol being apparently the most effective form for correction of hypovitaminosis D^45^.

Similarly, prospective and randomized studies in the dialysis population concluded that the administration of cholecalciferol and ergocalciferol was effective for the correction of hypovitaminosis D, but had no benefit for the control of SHPT^3^ ^,^ ^36^. Similar conclusions were observed in a meta-analysis published in 2011^37^. It is worth noting that these studies used large doses of cholecalciferol (10,000-200,000 IU weekly) for different follow-up periods (8-24 weeks), which may have interfered with the observation of some significant effect on PTH values and vascular calcification^47^ ^-^ ^50^. The dosages analyzed, even the higher ones, were not associated with drug toxicity phenomena^49^. In summary, most of the available data emerge from observational and some randomized studies, but with limited number of participants and with widely varying supplementation schemes. 

Finally, it is noteworthy that although the population on dialysis with low PTH values has progressively increased^51^, studies on vitamin D supplementation in this population are scarce. One option described would be the administration of low doses of cholecalciferol (25,000 to 50,000 IU monthly)^52^, on an individualized basis, with adequate monitoring and avoiding active forms of vitamin D, so as to avoid exaggerated suppression of PTH and vitamin D intoxication^3^ ^,^ ^51^. A consensus review was recently published with information regarding the prescription of cholecalciferol, based on serum 25(OH)D values, highlighting the importance of maintaining these levels > 30 ng/mL in the CKD population, with a recommendation not to exceed 60 ng/mL^53^. Table 3 presents a recommendation for cholecalciferol supplementation to correct hypovitaminosis D, based on serum 25(OH)D levels. 

**Table 3.** Guidelines for cholecalciferol supplementation in CKD

Level of 25(OH)D (ng/ml)	Cholecalciferol dose (IU)	Supplementation Time
< 5	50,000 IU/week for 12 weeks After 50,000 IU/month	6 months and new dosage
5-15	50,000 IU/week for 4 weeks After 50,000 IU/month	6 months and new dosage
16-30	50,000 IU/month	6 months and new dosage

## Nutritional vitamin D in kidney transplant patients (renal Tx)

Kidney transplant patients have impaired vitamin D metabolism, which is determined by graft function, FGF 23 and PTH levels, as well as by immunosuppressive therapy and other factors such as nutritional status and skin exposure to sunlight^54^. Hypovitaminosis D is common in kidney Tx patients, with prevalence ranging from 30-81%^55^, especially among patients of African descent and during the first year after renal Tx^56^. Habitual steroid use impairs the activation of enzymes that regulate vitamin D metabolism and favors the increase in PTH and FGF-23^57^. On the other hand, immunosuppressive regimens that avoid the use of corticosteroids determine improved vitamin D metabolism^55^. Additionally, the use of calcineurin inhibitors is associated with the presence of low vitamin D levels^58^, while the use of rapamycin does not seem to interfere with 25(OH)D metabolism^59^. Finally, some authors have observed that hypovitaminosis D may be associated with lower glomerular filtration rate (GFR) values over 12 months and increased risk of interstitial fibrosis and tubular atrophy^60^, especially when serum levels are < 12 ng/mL^54^. 

Although some authors report significant benefit in the control of mineral metabolism variables (reduction of PTH, improved bone health, and appropriate regulation of calcemia) with 25(OH)D3 supplementation in the transplanted population^61^ ^,^ ^62^, the effects of cholecalciferol and ergocalciferol supplementation remain controversial. Finally, the most recent guidelines suggest that vitamin D deficiency and insufficiency should be actively checked in the renal Tx population and corrected with cholecalciferol or ergocalciferol, following the same recommendations for the general population, given their positive effects on PTH control and for bone mass^10^. 

## References

[B1] Holick MF (2014). Sunlight, ultraviolet radiation, vitamin D and skin cancer: how
much sunlight do we need?. Adv Exp Med Biol.

[B2] Macdonald HM (2013). Contributions of sunlight and diet to vitamin D
status. Calcif Tissue Int.

[B3] Morrone LF, Bolasco P, Camerini C, Cianciolo G, Cupisti A, Galassi A (2016). Vitamin D in patients with chronic kidney disease: a position
statement of the Working Group "Trace Elements and Mineral Metabolism" of
the Italian Society of Nephrology. J Nephrol.

[B4] Bikle DD (2014). Vitamin D metabolism, mechanism of action, and clinical
applications. Chem Biol.

[B5] Dusso AS (2011). Kidney disease and vitamin D levels: 25-hydroxyvitamin D,
1,25-dihydroxyvitamin D, and VDR activation. Kidney Int Suppl(2011).

[B6] Andress DL (2006). Vitamin D in chronic kidney disease: a systemic role for
selective vitamin D receptor activation. Kidney Int.

[B7] Carvalho AB, Gueiros APS, Gueiros JE de B, Neves CL, Karohl C, Sampaio E (2012). Guidelines on bone mineral disorder in chronic kidney
disease--addendum chapter 2. J Bras Nefrol.

[B8] National Kidney Foundation (2003). K/DOQI clinical practice guidelines for bone metabolism and
disease in chronic kidney disease. Am J Kidney Dis.

[B9] Kidney Disease: Improving Global Outcomes (KDIGO) CKD-MBD Work
Group (2009). KDIGO clinical practice guideline for the diagnosis, evaluation,
prevention, and treatment of Chronic Kidney Disease-Mineral and Bone
Disorder (CKD-MBD). Kidney Int Suppl.

[B10] Kidney Disease: Improving Global Outcomes (KDIGO) CKD-MBD Update
Work Group (2017). KDIGO 2017 Clinical Practice Guideline Update for the Diagnosis,
Evaluation, Prevention, and Treatment of Chronic Kidney Disease-Mineral and
Bone Disorder (CKD-MBD). Kidney Int Suppl (2011).

[B11] Jean G, Souberbielle JC, Chazot C (2017). Vitamin D in chronic kidney disease and dialysis
patients. Nutrients.

[B12] LaClair RE, Hellman RN, Karp SL, Kraus M, Ofner S, Li Q (2005). Prevalence of calcidiol deficiency in CKD: a cross-sectional
study across latitudes in the United States. Am J Kidney Dis.

[B13] Barreto DV, Barreto FC, Liabeuf S, Temmar M, Boitte F, Choukroun G (2009). Vitamin D affects survival independently of vascular
calcification in chronic kidney disease. Clin J Am Soc Nephrol.

[B14] Bucharles S, Barberato SH, Stinghen AE, Gruber B, Meister H, Mehl A (2011). Hypovitaminosis D is associated with systemic inflammation and
concentric myocardial geometric pattern in hemodialysis patients with low
iPTH levels. Nephron Clin Pract.

[B15] Shah N, Bernardini J, Piraino B (2005). Prevalence and correction of 25(OH) vitamin D deficiency in
peritoneal dialysis patients. Perit Dial Int.

[B16] Boudville NC, Hodsman AB (2006). Renal function and 25-hydroxyvitamin D concentrations predict
parathyroid hormone levels in renal transplant patients. Nephrol Dial Transplant.

[B17] Mucsi I, Almási C, Deák G, Marton A, Ambrus C, Berta K (2005). Serum 25(OH)-vitamin D levels and bone metabolism in patients on
maintenance hemodialysis. Clin Nephrol.

[B18] Milinković NL, Majkić-Singh NT, Mirković DD, Beletić AD, Pejanović SD, Vujanić ST (2009). Relation between 25(OH)-vitamin D deficiency and markers of bone
formation and resorption in haemodialysis patients. Clin Lab.

[B19] Lee Y-H, Kim JE, Roh YH, Choi HR, Rhee Y, Kang DR (2014). The combination of vitamin D deficiency and mild to moderate
chronic kidney disease is associated with low bone mineral density and
deteriorated femoral microarchitecture: results from the KNHANES
2008-2011. J Clin Endocrinol Metab.

[B20] Lips P, Goldsmith D, de Jongh R (2017). Vitamin D and osteoporosis in chronic kidney
disease. J Nephrol.

[B21] Boudville N, Inderjeeth C, Elder GJ, Glendenning P (2010). Association between 25-hydroxyvitamin D, somatic muscle weakness
and falls risk in end-stage renal failure. Clin Endocrinol (Oxf).

[B22] Ahmadi F, Damghani S, Lessan-Pezeshki M, Razeghi E, Maziar S, Mahdavi-Mazdeh M (2016). Association of low vitamin D levels with metabolic syndrome in
hemodialysis patients. Hemodial Int.

[B23] Lai S, Coppola B, Dimko M, Galani A, Innico G, Frassetti N (2014). Vitamin D deficiency, insulin resistance, and ventricular
hypertrophy in the early stages of chronic kidney disease. Ren Fail.

[B24] London GM, Guerin AP, Verbeke FH, Pannier B, Boutouyrie P, Marchais SJ (2007). Mineral metabolism and arterial functions in end-stage renal
disease: potential role of 25-hydroxyvitamin D deficiency. J Am Soc Nephrol.

[B25] Fusaro M, Gallieni M, Rebora P, Rizzo MA, Luise MC, Riva H (2016). Atrial fibrillation and low vitamin D levels are associated with
severe vascular calcifications in hemodialysis patients. J Nephrol.

[B26] Wolf M, Shah A, Gutierrez O, Ankers E, Monroy M, Tamez H (2007). Vitamin D levels and early mortality among incident hemodialysis
patients. Kidney Int.

[B27] Patel NM, Gutierrez OM, Andress DL, Coyne DW, Levin A, Wolf ALM (2010). Vitamin D deficiency and anemia in early chronic kidney
disease. Kidney Int.

[B28] Isakova T, Gutierrez OM, Patel NM, Andress DL, Wolf M, Levin A (2011). Vitamin D deficiency, inflammation, and albuminuria in chronic
kidney disease: complex interactions. J Ren Nutr.

[B29] Pilz S, Iodice S, Zittermann A, Grant WB, Gandini S (2011). Vitamin D status and mortality risk in CKD: a meta-analysis of
prospective studies. Am J Kidney Dis.

[B30] Li YC (2010). Renoprotective effects of vitamin D analogs. Kidney Int.

[B31] Andrukhova O, Slavic S, Zeitz U, Riesen SC, Heppelmann MS, Ambrisko TD (2014). Vitamin D is a regulator of endothelial nitric oxide synthase and
arterial stiffness in mice. Mol Endocrinol.

[B32] Alvarez J, Wasse H, Tangpricha V (2012). Vitamin D supplementation in pre-dialysis chronic kidney disease:
a systematic review. Dermatoendocrinol.

[B33] Cardoso MP, Pereira LAL (2019). Native vitamin D in pre-dialysis chronic kidney
disease. Nefrologia.

[B34] Tripkovic L, Lambert H, Hart K, Smith CP, Bucca G, Penson S (2012). Comparison of vitamin D2 and vitamin D3 supplementation in
raising serum 25-hydroxyvitamin D status: a systematic review and
meta-analysis. Am J Clin Nutr.

[B35] Goldsmith DJA (2016). Pro: Should we correct vitamin D deficiency/insufficiency in
chronic kidney disease patients with inactive forms of vitamin D or just
treat them with active vitamin D forms?. Nephrol Dial Transplant.

[B36] Agarwal R, Georgianos PI (2016). Con: nutritional vitamin D replacement in chronic kidney disease
and end-stage renal disease. Nephrol Dial Transplant.

[B37] Kandula P, Dobre M, Schold JD, Schreiber MJ, Mehrotra R, Navaneethan SD (2011). Vitamin D supplementation in chronic kidney disease: a systematic
review and meta-analysis of observational studies and randomized controlled
trials. Clin J Am Soc Nephrol.

[B38] Alvarez JA, Law J, Coakley KE, Zughaier SM, Hao L, Salles KS (2012). High-dose cholecalciferol reduces parathyroid hormone in patients
with early chronic kidney disease: a pilot, randomized, double-blind,
placebo-controlled trial. Am J Clin Nutr.

[B39] Drechsler C, Verduijn M, Pilz S, Dekker FW, Krediet RT, Ritz E (2011). Vitamin D status and clinical outcomes in incident dialysis
patients: results from the NECOSAD study. Nephrol Dial Transplant.

[B40] Jean G, Lataillade D, Genet L, Legrand E, Kuentz F, Moreau-Gaudry X (2011). Impact of hypovitaminosis D and alfacalcidol therapy on survival
of hemodialysis patients: results from the French ARNOS
study. Nephron Clin Pract.

[B41] Krause R, Schober-Halstenberg H-J, Edenharter G, Haas K, Roth HJ, Frei U (2012). Vitamin D status and mortality of German hemodialysis
patients. Anticancer Res.

[B42] Dawson-Hughes B, Mithal A, Bonjour J-P, Boonen S, Burckhardt P, Fuleihan GE-H (2010). IOF position statement: vitamin D recommendations for older
adults. Osteoporos Int.

[B43] Bucharles S, Barberato SH, Stinghen AEM, Gruber B, Piekala L, Dambiski AC (2012). Impact of cholecalciferol treatment on biomarkers of inflammation
and myocardial structure in hemodialysis patients without
hyperparathyroidism. J Ren Nutr.

[B44] Del Valle E, Negri AL, Fradinger E, Canalis M, Bevione P, Curcelegui M (2015). Weekly high-dose ergocalciferol to correct vitamin D
deficiency/insufficiency in hemodialysis patients: a pilot
trial. Hemodial Int.

[B45] Daroux M, Shenouda M, Bacri J-L, Lemaitre V, Vanhille P, Bataille P (2013). Vitamin D2 versus vitamin D3 supplementation in hemodialysis
patients: a comparative pilot study. J Nephrol.

[B46] Gregório PC, Bucharles S, da Cunha RSD, Braga T, Almeida AC, Henneberg R (2021). In vitro anti-inflammatory effects of vitamin D supplementation
may be blurred in hemodialysis patients. Clinics.

[B47] Marckmann P, Agerskov H, Thineshkumar S, Bladbjerg E-M, Sidelmann JJ, Jespersen J (2012). Randomized controlled trial of cholecalciferol supplementation in
chronic kidney disease patients with hypovitaminosis D. Nephrol Dial Transplant.

[B48] Armas LA, Andukuri R, Barger-Lux J, Heaney RP, Lund R (2012). 25-Hydroxyvitamin D response to cholecalciferol supplementation
in hemodialysis. Clin J Am Soc Nephrol.

[B49] Wasse H, Huang R, Long Q, Singapuri S, Raggi P, Tangpricha V (2012). Efficacy and safety of a short course of very-high-dose
cholecalciferol in hemodialysis. Am J Clin Nutr.

[B50] Delanaye P, Weekers L, Warling X, Moonen M, Smelten N, Médart L (2013). Cholecalciferol in haemodialysis patients: a randomized,
double-blind, proof-of-concept and safety study. Nephrol Dial Transplant.

[B51] Bover J, Urena P, Brandenburg V, Goldsmith D, Ruiz C, DaSilva I (2014). Adynamic bone disease: from bone to vessels in chronic kidney
disease. Semin Nephrol.

[B52] Jansen RB, Svendsen OL (2014). The effect of oral loading doses of cholecalciferol on the serum
concentration of 25-OH-vitamin-D. Int J Vitam Nutr Res.

[B53] Moreira CA, Ferreira CEDS, Madeira M, Silva BCC, Maeda SS, Batista MC (2020). Reference values of 25-hydroxyvitamin D revisited: a position
statement from the Brazilian Society of Endocrinology and Metabolism (SBEM)
and the Brazilian Society of Clinical Pathology/Laboratory Medicine
(SBPC). Arch Endocrinol Metab.

[B54] Keyzer CA, Riphagen IJ, Joosten MM, Navis G, Kobold ACM, Kema IP (2015). Associations of 25(OH) and 1,25(OH)2 vitamin D with long-term
outcomes in stable renal transplant recipients. J Clin Endocrinol Metab.

[B55] Alshayeb HM, Josephson MA, Sprague SM (2013). CKD-mineral and bone disorder management in kidney transplant
recipients. Am J Kidney Dis.

[B56] Querings K, Girndt M, Geisel J, Georg T, Tilgen W, Reichrath J (2006). 25-hydroxyvitamin D deficiency in renal transplant
recipients. J Clin Endocrinol Metab.

[B57] Akeno N, Matsunuma A, Maeda T, Kawane T, Horiuchi N (2000). Regulation of vitamin D-1alpha-hydroxylase and -24-hydroxylase
expression by dexamethasone in mouse kidney. J Endocrinol.

[B58] Filipov JJ, Zlatkov BK, Dimitrov EP, Svinarov D (2015). Relationship between vitamin D status and immunosuppressive
therapy in kidney transplant recipients. Biotechnol Biotechnol Equip.

[B59] Westenfeld R, Schlieper G, Wöltje M, Gawlik A, Brandenburg V, Rutkowski P (2011). Impact of sirolimus, tacrolimus and mycophenolate mofetil on
osteoclastogenesis--implications for post-transplantation bone
disease. Nephrol Dial Transplant.

[B60] Bienaime F, Girard D, Anglicheau D, Canaud G, Souberbielle JC, Kreis H (2013). Vitamin D status and outcomes after renal
transplantation. J Am Soc Nephrol.

[B61] Courbebaisse M, Thervet E, Souberbielle JC, Zuber J, Eladari D, Martinez F (2009). Effects of vitamin D supplementation on the calcium-phosphate
balance in renal transplant patients. Kidney Int.

[B62] Wissing KM, Broeders N, Moreno-Reyes R, Gervy C, Stallenberg B, Abramowicz D (2005). A controlled study of vitamin D3 to prevent bone loss in
renal-transplant patients receiving low doses of steroids.
Transplantation. Transplantation.

